# Health service utilization for low back pain in Germany between 2000 and 2020—a scoping review of claims data

**DOI:** 10.3389/fpain.2025.1661722

**Published:** 2025-09-16

**Authors:** Julia Truthmann, Simone Kiel, Georg Vrana, Jean-François Chenot

**Affiliations:** Department of General Practice, Institute for Community Medicine, University Medicine Greifswald, Greifswald, Germany

**Keywords:** scoping review, claims data, health service utilization, low back pain, Germany

## Abstract

**Background:**

Low back pain (LBP) uses a large proportion of health care resources. Data are needed for health care planning, measuring adherence to guidelines for quality assurance, and assessing overuse and underuse of health care services. The aim of this review is to summarize claims data and describe trends in health care utilization for LBP for the years 2000 to 2020.

**Methods:**

This scoping review summarizes studies and health reports using claims data of people aged ≥15 years covered by a statutory health insurance in Germany for the period 2000 to 2020. We searched publications in PubMed, EMBASE and Google. Data on health care services were extracted and trends over the years were summarized.

**Results:**

We included data from 76 publications, health reports and online databases. Every year, 25 to 32% of adults in Germany seek care for LBP. Most of the claims data cannot be pooled because of differences in standardization and reporting. However, trends are observable. Magnetic resonance imaging increased to 7.5%, plain radiography decreased to 15%. The number of sick leave days decreased slightly over time. Hospital admissions for LBP, spinal surgery, and opioid use increased. Outpatient rehabilitation increased, but the overall use of rehabilitation services remained relatively stable.

**Conclusions:**

Inconsistent reporting standards and fragmentation of German claims data reporting, hinders a comprehensive understanding of health service utilization for low back pain. Despite limitations, current data suggest potential overuse of resources for LBP in Germany, consistently with international data. Given the high proportion of patients consulting for LBP better monitoring of health service utilization is needed to improve quality of care and resource allocation.

## Background

Low back pain (LBP) is a major health care problem worldwide, responsible for substantial medical and economic use of resources (1, 2). The most commonly prescribed treatments are drug treatment, exercise, massage therapy and spinal manipulation (3). An increase in health service utilization for LBP has been described for many industrialized countries (3, 4). Paradoxically, this has not led to a decrease in disease burden (4). A systematic review of population-based observational studies for LBP, showed wide regional variations in Europe and a significant proportion of people with LBP, who do not seek health-care (3). Notably, the study did not include German data because most relevant claims data were only published as grey literature, limiting standardized data extraction and analysis. Access to such data is needed for health policy, health care planning, measuring guideline adherence for quality assurance, assessing over- and underuse of health care services, and monitoring trends. Health care costs are expected to increase in industrialized countries over time due to an aging population. In addition, recent studies have shown that substantial health care expenditures for LBP are due to overuse (5–7), which may change over time due to e.g., advances in imaging or changes in guideline recommendations.

In Germany, universal health insurance is provided for approximately 90% of the population by various statutory health insurances (SHI) (8). Germany currently has about 95 SHI providers as of 2025. All these SHIs operate under uniform legal frameworks, with minor differences in supplementary services, contributions, or member benefits. The two biggest are “Techniker Krankenkasse (TK)” and the collective “AOK” system, followed by “BARMER” and “DAK Gesundheit”. Most SHIs publish health reports on various health issues based on their members’ data. The comparison of data is limited due to demographic differences between the members of various SHI. Comprehensive data on health service utilization for LBP in Germany has not been published in the last 10 years and is mostly based on surveys of relatively small samples (9). Until recently, claims data analysis of health care data for epidemiological or research purposes was limited due to data protection regulations and legal concerns. Within the last 10 years, aggregated data on health care utilization from SHIs became increasingly available.

A health report based on data of the SHI “Barmer” (10) showed that, many of the treatments provided in 2005 had low or unclear evidence based on the European guidelines for the management of acute non-specific LBP in primary care (11). From an economic point of view, 22% of the total expenditure, about €8 million, was spent on ineffective treatments.

The aim of this review is to identify sources of claims data on health care utilization for LBP in Germany, to summarize the data and to describe trends in health care utilization for the period 2000 to 2020.

## Methods

### Literature and data search

This scoping review is reported following the standards of reporting using the PRISMA-checklist for Scoping Reviews (PRISMA-ScR). We did not register the review in advance. A PubMed and EMBASE literature review of published German claims data on health care utilization for LBP was conducted. Since most health care data is published in health reports from SHI's in Germany, which are not listed in medical databases, additional non-systematic web-based searches in Google Scholar und Google were performed. Since not all health reports we were aware of, were available online, the following SHI were contacted for additional health reports: AOK, BKK, Barmer GEK and TK. The study outcomes are defined in Table 1.

**Table 1:** Definition of outcomes.

Health services	Specification
Ambulatory consultation	Coded diagnoses (ICD-10 codes)
Imaging	Billing codes for x-ray, computed tomography (CT), magnetic resonance imaging (MRI)
Non-pharmaceutical prescription treatments	Billing codes for exercise therapy, manual therapy, heat or cold application, massage, and traction
Non-invasive therapies	billing codes for Manual therapy and acupuncture, when provided by a physician
Opioid prescription	Opioid therapy, mainly based on ATC codes
Sick leave days	Days of sick leave among the working population
Hospitalization and days spent in hospital	DRG codes
Spinal surgery	OPS codes
Rehabilitation for chronic LBP	Inpatient and outpatient rehabilitation

OPS, operations, procedures and general medical measures [German Procedure Classification].

### Eligibility criteria

We included publications on German claims data of people aged ≥15 years, reporting health care utilization for the years 2000 to 2020 in English or German. Claims data are defined as data collected for administrative or claims purposes. We considered the ICD-10 codes M42 (spinal osteochondrosis), M47 (spondylosis), M48 (Other spondylopathies), M51 (other intervertebral disc disorders), M53 (Other dorsopathies, not elsewhere classified), M54 (dorsalgia) and M99 (biomechanical lesions, not elsewhere classified). M50 (cervical disc disorders) was included if only aggregated data were available.

### Search methods and data extraction

The literature search was conducted in September 2019 and updated in April 2025. We present the search strategy for each database in Additional File 1. We merged the list of publications using Excel and manually removed duplicates. Two reviewers (SK, GV) independently screened publications for inclusion in the review. A third reviewer (JFC) was available to resolve disagreement if needed. Full-text screening and data extraction was done by one reviewer (GV). We extracted data from each publication using Excel. We also took note of the observation period, if data in the original publications were standardized according to the population structure or not, the specific ICD-10 codes and if available the regional differences. Wherever possible, we displayed data of different sources together in one figure. Extracted values have been rounded to full percentages.

## Results

### Included health reports, online databases and studies

**Figure 1:**
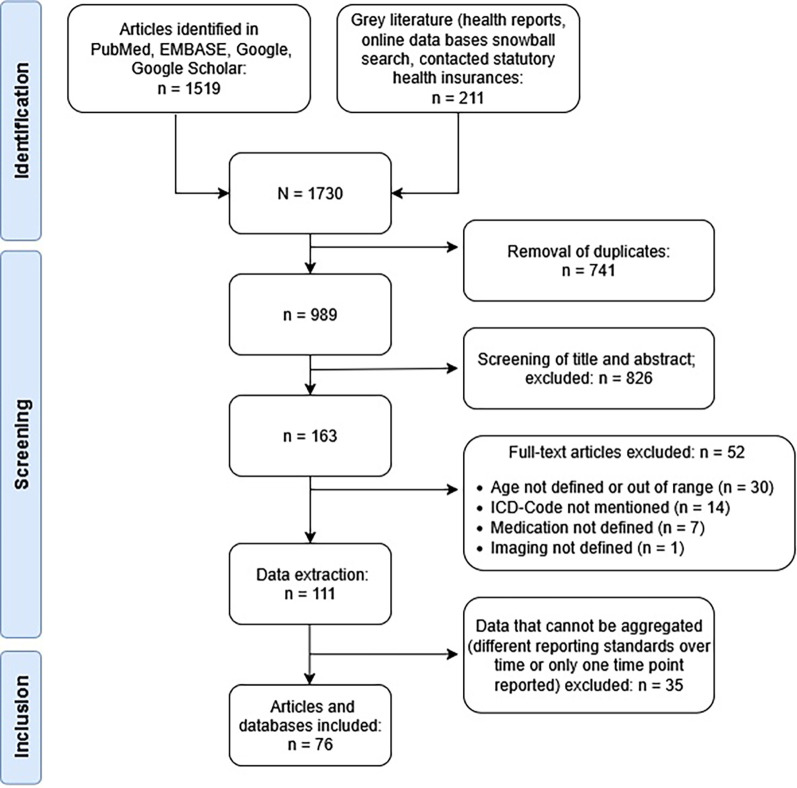
Study flow chart.

Figure 1 provides details on the search and selection process. A total of 1,519 publications, 211 health reports and online data bases using German claims data were found. After removal of duplicates, 989 articles were screened, 826 were excluded, because they did not meet the inclusion criteria. Full-text screening was done for 163 articles, 52 were excluded. The review includes data from a total of 76 publications (Additional File 1). Data from 35 publications could not be aggregated because of different reporting standards or because only one time point was reported. Data on rehabilitation services which are provided by the German Pension Insurance for people of working age are published in online databases (12–14).

Table 2 shows the different publication types included and the ICD-10 codes reported. Table 3 presents the health service outcomes and the source from which the data were extracted. An overview over standardization of extracted data from health reports of statutory health insurances, federal health monitoring, federal statistical office and single publications is provided in Additional File 2.

**Table 2:** ICD-Codes used to define low back pain in health reports and single publications.

Articles included	M54	M53	M51	M50^b^	M47	M48	M42	M99	M00–M99
Health reports of statutory health insurances									
BKK	*n* = 8	x	x^a^			x			
DAK	*n* = 11	x	x^a^	x					
TK	*n* = 17	x	x^a^	x					
Barmer/GEK	*n* = 23	x	x	x		x			
AOK	*n* = 5	x	x	x		x^a^	x		
Online data bases									
Federal Health Monitoring (12, 13)	x^b^	x^b^	x^b^	x^b^	x^b^	x^b^	x^b^		x
Federal Statistical Office (14)	x^b^	x^b^	x^b^	x^b^	x^b^				
Single publications									
DEWI project (15–17)	x	x	x	x	x	x	x		
Zich & Tisch (18)	x		x		x	x			
Höer et al. (19)	x		x		x	x			
Hickstein et al. (20)	x	x			x				
Chenot et al. (21)	x				x		x		
Andersohn et al. (22)			x		x	x	x		
Walker et al. (23)	x							x	
Kasbohm et al. (24)	x	x	x		x	x	x		

^a^ Not found in every health report.

^b^ For inpatient rehabilitation only.

M54 (dorsalgia), M53 (other dorsopathies, not elsewhere classified), M51 (other intervertebral disc disorders), M50 (cervical disc disorders), M47 (spondylosis), M48 (other spondylopathies), M42 (spinal osteochondrosis), M99 (biomechanical lesions, not elsewhere classified), M00-M99 (diseases of the musculoskeletal system and connective tissue).

**Table 3:** Sources of extracted data.

Health services	Health reports of statutory health insurances					Online data bases	Single publications
	BKK	DAK	TK	BARMER/GEK	AOK		
Ambulatory consultation		x			x		x
Imaging				x			x
Invasive non-operative therapies				x			x
Non-pharmaceutical prescription treatments				x	x		x
Non-invasive therapies				x	x		x
Prescription medication				x			x
Sick leave days	x	x	x	x	x		
Hospitalization	x	x		x			x
Spinal surgery				x			x
Rehabilitation	x			x		x	

**Figure 2:**
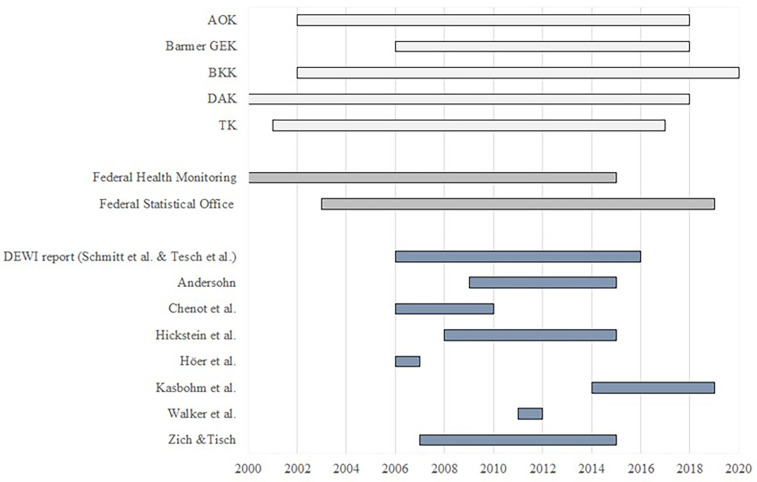
Overview of observation periods. Health insurance reports (light grey), online data bases (grey) and other (peer reviewed) publications (blue).

Figure 2 gives an overview over the observation periods of extracted data from each health report of SHI, online data bases and from publications. In 2010, the SHI Barmer merged with the SHI GEK and therefore includes data between 2006 and 2010 from SHI Barmer only.

### Ambulatory consultations for LBP

Due to different standardization methods and ICD-10 codes reported, data on ambulatory consultations cannot be summarized visually. In 2010, 32% of employed persons aged 15–65 years were diagnosed with M54 (dorsalgia) and 10.0% with M53 (Other dorsopathies, not elsewhere classified) (25). The frequency of consultations in ambulatory care for patients diagnosed with M54 increased with age, and more women than men sought ambulatory medical care (26). In 2016, 23% of DAK insured persons were in outpatient treatment for M54 (dorsalgia) and 6% for M53 (27). In the time period from 2011 to 2016, the outpatient diagnosis M54 remained relatively constant at 23%–25% (27). In total, the consultation rate in 2010 was 26% within one year, including outpatient and inpatient consultations for M42 (spinal osteochondrosis) to M54 (dorsalgia) (21). It is assumed only a few patients (<1%) are treated as inpatients, therefore the frequency of consultations in this health report applies more to outpatient care (21). Again, more women (28%) than men (25%) tend to seek medical care (21).

The Bertelsmann Foundation analyzed data from 7 million statutory health insured people and considers M54 and M99 (biomechanical lesions) (22). From 2009 to 2015, a slight increase in number of consultations for LBP can be observed (433/1,000 insured people to 469/1,000 insured people) (22). Of those, 43% of patients consulted a doctor once, 18% twice, 12% three times and 28% ≥ 4 times.

In Mecklenburg-Western Pomerania (24) each year, about 38% of adults receive a LBP diagnosis (M40-M54). In 2019, 76% of ICD-10 codes were billed by GPs, 16% by orthopaedists, and 7% by neurologists and neurosurgeons.

### Imaging for LBP

**Figure 3:**
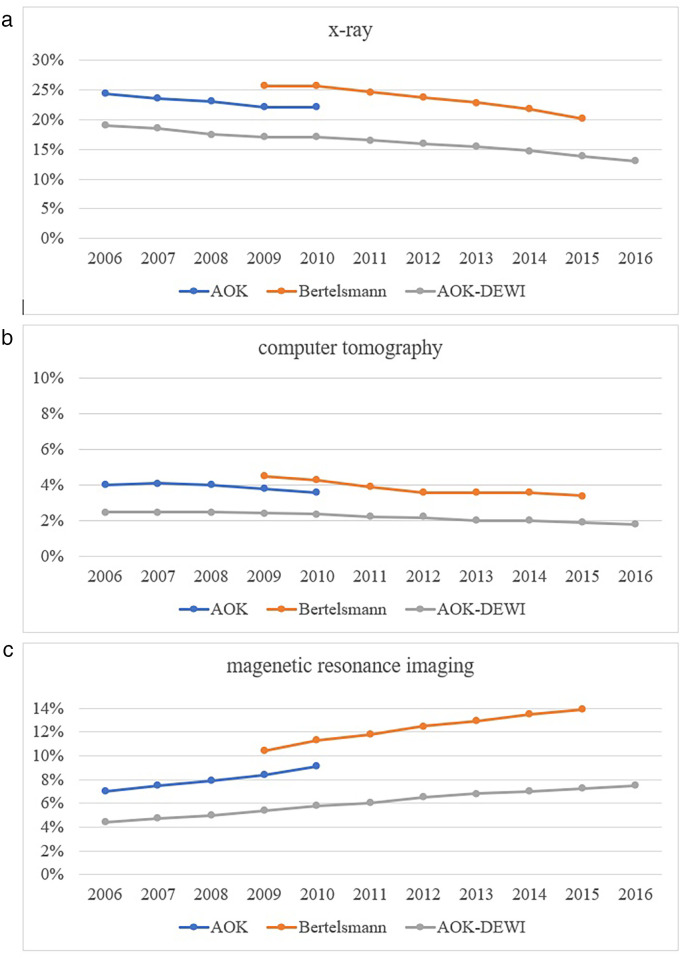
**(a–c)** time trend in the proportion of patients receiving imaging for low back pain.

The frequency of imaging (x-ray, computed tomography (CT), magnetic resonance imaging (MRI)) for LBP was reported for the period 2006–2015 (15, 21, 22). They reported a decrease in conventional imaging with x-ray and CT and a continuous increase of MRI (Figures 3a–c). Slight deviations exist due to different ICD-10 codes and inclusion criteria. The report from the Bertelsmann Foundation reports subgroup-analyses for 2015, where a total of 37% consulting for LBP received imaging, with 58% of those with coded red flag pathologies and 16.4% without. Women received slightly more often imaging (39% vs. 36%). The proportion of patients receiving imaging consulting a GP only for LBP was 22% compared to 58% seen only by an orthopedic surgeon. In patients consulting both, the proportion was 84%. Similar differences between patients treated by GP only and patients additionally treated by other specialists were described in a population based cohort study (24). An analysis of patients admitted to hospital for LBP shows a higher proportion of imaging (x-ray 46%, CT 12.6%, MRI 4.5%) (28). The data from Schmitt et al. cannot directly be compared due to various inclusion and exclusion criteria, but confirm the trend (15). Analyzing timing, 21%, received imaging within 4 weeks, 29%after 6 weeks and 46% after 24 weeks, interpreted as potential overuse (29). This is in line with the findings of the Bertelsmann Foundation where 22%–25% of patients with LBP received imaging within the 3 months claims period (22).

### Non-pharmaceutical prescription treatments for LBP

**Figure 4:**
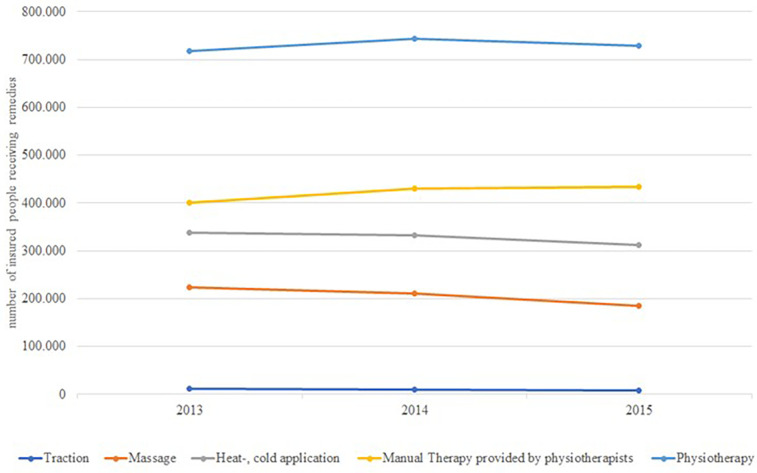
Time trends of the utilization of remedies (prescription physiotherapy and physical therapy) by all insured people of the barmer GEK.

Non-pharmacological treatments that are billable include physiotherapy, delivered as exercise therapy, manual therapy, heat or cold application, massage, and traction. Exercise therapy is most often prescribed followed by heat or cold application and massage (21). Another report from Barmer GEK summarized data from 2013 to 2014 on spinal disorders (30). These data are summarized in Figure 4. Again, exercise therapy was prescribed most frequently and it remained relatively constant over the two-year observation period. The prescription of manual therapy provided by physiotherapists increased slightly from 2013 to 2014, while massage, heat or cold application decreased slightly (21, 30). Traction remained constant throughout the two-year period (30). Another study described a slight overall increase of 3.0% comparing prescription rates for all physiotherapy treatments from 2010 to 2016 (16). Exercise therapy (+7.5%) and manual therapy (+43.6%) increased and massage decreased (−42.7%) (16). Data of the SHI AOK for the year 2015 showed that 47% of all prescribed massages were due to M54 (dorsalgia). Followed by manual therapy with 36% and exercise therapy with 18% (31). In 2016, the proportion of prescribed massages was 50% (32). The prescription of manual therapy for M54 (dorsalgia) remained stable at 36% in 2016 compared to the previous year. However, it dropped to 34% in 2018 (33, 34). The proportion of exercise therapy attributable to M54 (dorsalgia), remained the same at 18% in 2016, and decreased slightly to 17% in the two following years (32–34).

### Non-invasive therapies

Manual therapy and acupuncture, when provided by a physician, were considered as non-invasive therapies. In Germany, claims privileges for manual therapy require a qualification. While the majority of orthopedists is qualified, only a small percentage of GPs are able to bill manual therapy. The Barmer GEK hospital report (35), the health care report 2013/2014 (21), a report on the DEWI project (16) and two peer reviewed studies (20, 23) included data on those therapies. Data could not be aggregated due to different definitions of the insured persons, reported ICD-10 codes and lack of uniform standardization.

A study, analyzing claims data from 84 SHIs, covering approximately 7 million people in Germany, reports that 18% of patients with acute LBP (M54, dorsalgia) received manual therapy from physicians (23). The proportion of LBP patients (M42 (osteochondrosis), M47 (spondylopathy, M48, (other spondylopathies) M51 [intervertebral disc disorder, M54 (dorsalgia)] who were treated with manual therapy remained stable at 22%–23% from 2006 to 2010 (21).

The Barmer GEK hospital report 2015 (35) provides data on the treatment process during hospital stays in 2013. In the 365 days prior to hospitalization, manual therapy was received by 36% (35). With regard to the main diagnoses associated with hospitalization, 36% of patients were diagnosed with M48 (other spondylopathies), 39% with M51 (intervertebral disc disorders), and 34% with M54 (dorsalgia) (35).

The health care report 2013/2014 shows the proportion of LBP patients treated with acupuncture remain stable at 6% from 2007 to 2010 (21). A retrospective observational study reported a 7% utilization of acupuncture in 2014 (20). The most prevalent diagnosis among all acupuncture patients was M54 (dorsalgia) (51%). A significant sex disparity, with women accounting for double the number of acupuncture treatments was reported (20). Furthermore, from 2010 to 2015, the number of insured persons treated with acupuncture for knee or low back pain decreased from 7.5 to 6.5% (20). Another study showed an decrease of acupuncture from 2010 to 2016 by 20% (16).

### Opioid prescription

Four health reports (16, 19, 21, 35) included data on opioid use. One report based on AOK data showed that in 2010, approximately 11% of patients with LBP received low potency opioids and 2.7% high potency opioids (21). There was an 32% increase in high potency opioid prescriptions for LBP from 2006 to 2010. A report based on AOK data found that low and high potency opioids were prescribed to 5.7% and 1.9% of insured persons in 2010 (16). While prescriptions for low potency opioids decreased by 2.3% by 2016, prescriptions for high potency opioids increased by 19.0%. Another report analyzing data from 2006 to 2007 found that 25% of patients with LBP received at least one prescription for opioids (19). An analysis by Barmer GEK reported that 40% of patients admitted to the hospital for LBP had received an opioid prescription in the year before admission (35).

### Sick leave days for LBP

**Figure 5:**
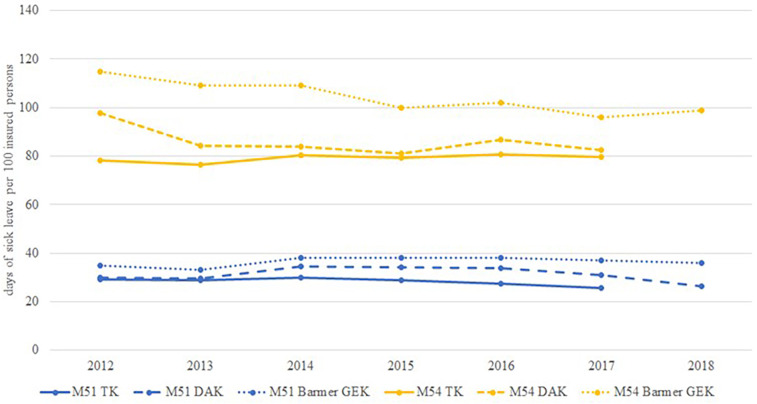
Time trend of sick leave days per 100 insured persons due to back pain (M51 and M54).

Since 2012 the data from the SHI TK (36–40), DAK (27, 41–45) and Barmer GEK (46–50) have been standardized for age and sex, based on the structure of Germany's working population in 2010. Previous health reports on sick leave days were not comparable due to different analysis methods and inconsistent reporting. Figure 5 shows the temporal trend of the duration of sick leave due to LBP (M51—other intervertebral disc disorders, M54—dorsalgia) between 2012 and 2018. The number of sick leave days for M51 varied between 29 and 38 days/100 insured persons, and. remained at the same level for all three insurances. In 2012, the number of sick leave days for M54 varied between 78 days and 115 days/100 insured persons. For DAK and Barmer GEK, the number of sick leave days for M54 decreased slightly and was 83 days/100 insured persons for DAK in 2017 and 99 days/100 insured persons for Barmer GEK in 2018.

### Hospitalization and days spent in hospital

**Figure 6:**
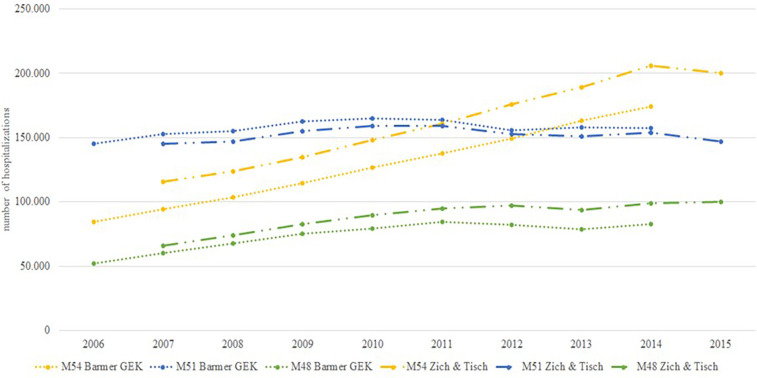
Time trend of hospitalizations due to back pain (M48, M51 and M54).

Health reports of the SHI BKK (2001–2010), DAK (2018), and GEK (28, 35, 51–69) as well as peer-reviewed publications (18, 21) showed an increase of hospitalizations for M51 (other intervertebral disc disorders), M54 (dorsalgia) and M48 (other spondylopathies) (28, 35, 51–69) (Figure 6). Overall (main diagnosis M47, M48, M51, M54), hospitalization for LBP increased by 30% between 2007 and 2015 (18). In 2015, 489,000 people were hospitalized due to LBP. In 2013, the majority of admissions with a main admission diagnosis of back pain (∼70%) was not related to spinal surgery (35). Other reasons for hospital admission include invasive pain therapy (∼30%), multimodal pain therapies (∼5%).

M54 was the most frequent diagnosis. The incidence rate of M54 (dorsalgia) hospitalizations rose from 10.3 cases/10,000 insurance years in 2006 to 20.2 cases/10,000 insurance years in 2014, representing a substantial increase of 95% (35). Another publication reported an 73% increase in hospitalizations for main diagnosis M54 from 2007 to 2015 (18). An analysis by DAK showed a lower increase of 31% for M54 from 2010 to 2016 (27). Incidence rates of other musculoskeletal diagnoses also showed a more modest increase. The number of hospitalizations for M47 increased by 31% and for M51 by 2% from 2007 to 2015 (18). There were strong regional differences in the number of hospitalizations for M54. The lowest rate of hospitalizations with 135/100,000 residents in 2014/2015 was reported in Hamburg, whereas 400/100,000 residents were reported for Saxony-Anhalt for the same time (18).

On average, patients with the diagnosis M54 (dorsalgia) spent 8 days in hospital, whereas patients with the diagnosis M51 (other intervertebral disc disorders) spent 9 days and M48 (Other spondylopathies) 10 days in hospital during 2005–2012 (61–67). From 2013 till 2017 it decreased to 7 days for the diagnoses M54 and M51 and to 9 days for the diagnosis M48 (28, 35, 60, 68, 69).

### Spinal surgery

**Figure 7:**
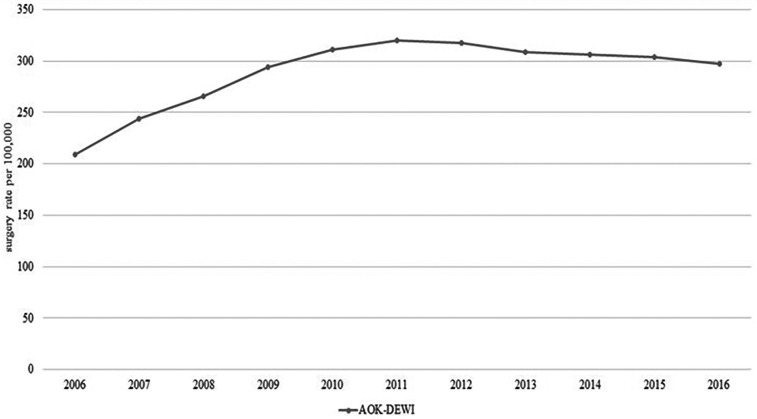
Time trend of spinal surgery (M40–54).

Four publications included data on spinal surgery (15, 17, 18, 35) based on the codes of the Operation and Procedure Classification System (OPS). Although patients diagnosed with M54 (dorsalgia) represent the majority of patients hospitalized with back pain, only 1.8% underwent spinal surgery (35). Patients who underwent surgery most commonly had the diagnosis M48 (Other spondylopathies) or M51 (other intervertebral disc disorders) (35). There was a 71% increase in the number of coded spinal procedures (OPS 5–83) from 452,000 to 772,000 between 2007 and 2015 (18). As several OPS codes are usually coded per operation, the increase does not directly correspond to the increase in the number of cases. Considering selected OPS codes showed an increasing number of procedures, e.g., excision of diseased intervertebral tissue (OPS 5–831) increased by 9%, spondylosis (OPS 5–836) increased by 56%, and bony decompression of the spinal canal (OPS 5–839.6) increased by 130% (18). Figure 7 shows the surgery rate per 100,000 insured persons from 2006 to 2016 based on AOK data (15, 17), suggesting an increase of 43%. Pronounced regional differences in the rate of spinal surgery were observed (15). There was a relative increase in coded procedures including OPS 5–831, 5–836, and 5–839.6 between 2007 and 2015 of 67% in Hesse and 66% in Thuringia, compared with 17% in Saxony-Anhalt (18). Schmitt et al. described that a high regional rate of spinal surgery is associated with a high number of MRI examinations as well as the surgical capacity of regional hospitals (15).

### Rehabilitation for chronic LBP

**Figure 8:**
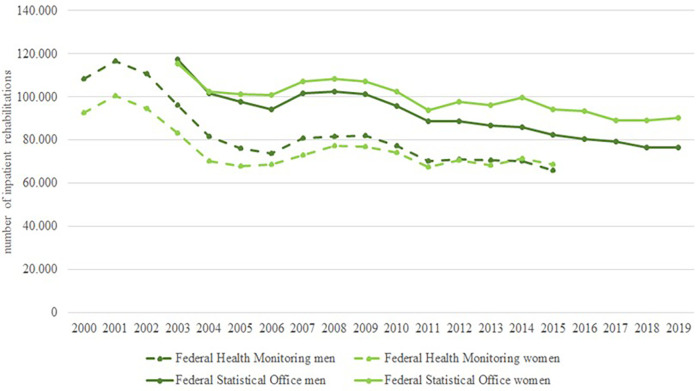
Time trend in inpatient rehabilitations with main diagnosis M50 to M54.

Rehabilitation for LBP aims to prevent disability and restore physical functioning to enable return to work. In Germany rehabilitation is mostly covered by the German pension insurance and not the SHI. Data on inpatient and outpatient rehabilitation for LBP are available in the online databases of the Federal Health Monitoring and the Federal Statistical Office (12–14). The SHI BKK also reported data on rehabilitation for pensioners and people in need of long-term care (51–55). Figure 8 shows the temporal trend of outpatient (8) and inpatient (9) rehabilitations with main diagnosis M00 to M99 (diseases of the musculoskeletal system and connective tissue) reported by the Federal Health Monitoring System. Only aggregated data for outpatient rehabilitations are available. Inpatient rehabilitation decreased from 344,000 cases in 2001 to 253,000 cases in 2004 (70). Since 2006, the number of inpatient rehabilitations has risen slightly to 262,000 in 2015. Outpatient rehabilitations with the main diagnosis M00—M99 (diseases of the musculoskeletal system and connective tissue) have steadily increased from 15,000 in 2001 to 83,000 in 2015.

**Figure 9:**
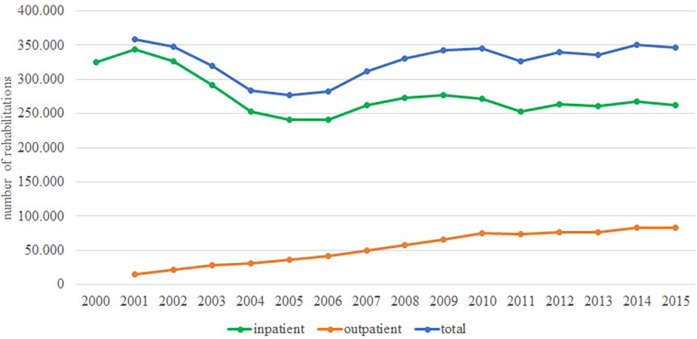
Time trend in inpatient and outpatient rehabilitations with main diagnosis ICD10-M00-99.

Figure 9 presents the temporal trend of inpatient rehabilitations with main diagnosis M50 to M54 (diseases of the spine) from 2000 to 2019. The data of the Federal Health Monitoring System include information on all employees subject to compulsory insurance, rehabilitation benefits of privately insured self-employed persons are not included (9). The data of the Federal Statistical Office include information on all insurance providers, but only from preventive care or rehabilitation facilities with more than 100 beds (14). There is a clear decline between 2001 and 2004. After 2004, both data sources show a slight decrease in inpatient rehabilitations. The decline is more pronounced for men than for women (35).

## Discussion

### Ambulatory consultations for LBP

While ICD-10 codes are used for billing purposes, they may not accurately reflect the true prevalence of LBP due to the incomplete coverage of related conditions, and potential biases in physician coding practices, which are influenced by individual, regional, and economic factors (24, 71). The health reports included various ICD-10 codes related to LBP which could not be pooled. Ambulatory consultations for LBP remained stable or increased slightly during the observation period, depending on the ICD-10 code. A total of 25% to 32% of all patients consulting ambulatory care are coded with a LBP diagnosis, mostly M54 (dorsalgia) (72). This mandates a step-wise and rational approach managing LBP. The finding that the frequency of ambulatory care consultations increased with age, and that more women than men sought ambulatory care is in line with international research (2). Most patients with the diagnoses M54 or M99 (biomechanical lesions) consulted a doctor once (42.7%). However, 27.6% of patients consulted four or more times, indicating chronic LBP An important reason to consult for LBP is the need to obtain a sick leave certificate. It has been suggested that consultation rates could be reduced by self-certification or sickness for longer periods (73). A systematic review estimated high rates of physician and ambulatory visits for LBP across high-income countries, but found wide variation depending on geography and health system. Reported rates ranged from 47% of patients in the UK and Europe, 67% in the USA, to 61.2% in Spain utilizing health-care services for LBP within a year (74). Comparing data across countries may help to contextualize national prevalence rates. However, it is not possible to make direct comparisons between the data presented in this review by summarizing all the ICD-10 codes related to LBP, since patients with LBP may receive more than one ICD-10 code.

### Sick leave days

Data show a slight decrease in the number of sick leave days between 2012 and 2018, which is due to a decrease in billed diagnoses of M54 (dorsalgia) (Figure 5). As data on the temporal trend of other diagnoses like M53 (unspecified dorsopathies), M47 (spondylopathies) are missing, it is not possible to conclude whether the data reflect a slight decrease in sick leave or if the observation is due to changes in billing behavior of physicians or to the increase in MRI, which is probably associated with claims of more specific LBP codes. In the Netherlands, the number of sick leave days decreased from about 9,000,000 days in 2002 to 6,000,000 days in 2007 (75). Interestingly, a study analyzing data from a primary care practice network in UK also found a decreasing trend in sickness certificates (76). The authors argued that this finding may be due to changes in physicians' behavior, as most guidelines encourage individuals to stay active and continue working despite pain. It is known that external factors such as the unemployment rate also influence sick leave (77). The increase in sick leave days for mental health disorders observed in the last decade in Germany is also a likely explanation (78).

### Imaging for LBP

National and international guidelines recommend imaging only in the presence of red flags within 4 to 6 weeks, depending on the urgency (79, 80). Approximately 16%–37% of all patients consulting for LBP receive some form of imaging depending on the setting (21, 22). The proportion of patients consulting for LBP, who receive imaging exceeds the proportion of patients expected to have a relevant pathology (∼1%). x-ray and CT imaging decreased while MRI increased. In 2015, about 15% of LBP patients received x-ray, 2.5% CT and 7.5% MRI. This is consistent with a meta-analysis that included data from the UK, Australia and the USA (81). This analysis found that approximately one-quarter of patients who presented to primary care were referred for imaging, and that one-third of patients who presented to emergency care were imaged. Overuse of imaging is observed in many industrialized countries (82, 83). Imaging is partly driven by the need to exclude serious pathologies and to avoid litigation. Analysis of the time from initial consultation to imaging shows that the majority of imaging is performed during a period in which most patients spontaneously improve (29). Imaging is an appropriate and measurable target included in most clinical indicators to monitor health care for LBP (84). However, there is a lack of effective interventions to reduce imaging for non-specific LBP (83).

### Non-pharmaceutical prescription treatments and non-invasive therapies for LBP

Data on physiotherapy services are sparse, so we cannot rule out changes in prescription practices. Exercise therapy is the most common non-pharmacological treatments for LBP. Approximately 20%–40% of patients are prescribed exercise therapy or manual therapy (spinal manipulation) by physiotherapists (31–34, 85). The German guideline for non-specific LBP recommends exercise therapy, and advises against massage therapy (80). A reduction in massage prescription has been observed, but was compensated with other forms of physiotherapy (16, 21, 30). The measurement of guideline adherence is constrained by limitations in ICD-10 coding. To control prescriptions for physiotherapy services, physicians have to manage a budget and receive feedback reports. Refusal to prescribe massage in particular is a source of patient dissatisfaction (86). Access to physiotherapy is increasingly restricted by a shortage of physiotherapists.

The effectiveness of manual therapy remains controversial (80). As an alternative to manual therapy provided by physiotherapists, physicians qualified in manual medicine, most orthopedic surgeons and about 15% of primary care providers, can provide and bill for manual therapy. It is estimated that manual therapy provided by physician is billed for ∼20% of patients with LBP (21, 23). A study comparing patients who received manual therapy with patients who did not receive manual therapy found no effect on sick leave, prescription of physiotherapy or pain medication (23).

Acupuncture is a popular but controversial treatment option for chronic LBP covered by SHI. It is estimated that ∼10%–20% of patients with chronic LBP receive acupuncture provided from physicians (20, 21). Acupuncture was included into the SHI services for patients with chronic LBP in 2007 following the German acupuncture study (87). The German guideline for non-specific LBP makes only an optional recommendation (80). The number of insured people with LBP treated with acupuncture has decreased (20). One explanation for the decline may be the integration of acupuncture in the standard service volume in 2010, which resulted in limited supplementary revenues benefits for physicians licensed to provide acupuncture.

### Opioid prescriptions

There are many reports on the use of prescription pain medications in Germany, but most are not limited to prescriptions related to LBP (88). Opioids are considered as a last resort for chronic LBP but the effectiveness of opioids has been questioned. National guidelines insist on careful evaluation of the individual clinical effectiveness of opioids for non-cancer pain (89). Several studies have shown an increase in the use of opioids for LBP in Germany (19, 21, 35). Approximately 10%–20% of patients with LBP receive low potency or high potency opioids (21, 37). From 2006 to 2010, there was an increase in prescriptions of scheduled opioids. There is no consensus on what is considered an appropriate or inappropriate proportion of patients with LBP receiving opioids. Germany is not facing an opioid crisis, but careful monitoring of prescribing patterns including long-term opioid prescribing for LBP, is warranted (89, 90). Opioid prescriptions for LBP are a suitable and measurable target for monitoring quality of care (84).

### Hospitalization and days spent in hospital

The number of hospitalizations for LBP increased steadily and doubled during the observation period (Figure 6). The increase in hospitalization rates can only be partially explained by demographic changes in the insured population. Most hospital admissions for non-surgical treatment of LBP are considered inappropriate, as LBP is considered an ambulatory care-sensitive condition (91). Germany has an extremely high number of hospital beds (800/100,000 inhabitants) compared with the OECD average (473/100,000 inhabitants) and a long length of stay (8.9 days) compared to the OECD average (7.4 days) (92). Data suggest that the length of stay has slightly decreased. Difficulties in accessing outpatient care, unmet patient needs, complications of treatment and inappropriate use of emergency departments are the most likely factors contributing to the observed increase in hospital care (27, 92).

### Spinal surgery

Clinical guidelines do not recommend surgery for non-specific LBP. Surgical interventions are generally only considered in specific cases, such as severe or progressive back or leg pain that is unresponsive to other therapies, or when there are red flags. Nevertheless, an increase in spinal surgery has been observed in Germany and internationally (93–95). It also can only be explained to a limited extent by the demographic change of the population. The observed regional heterogeneity in spinal surgery rates could not be explained by adjustment for confounding factors (17). This suggests that unrelated factors such as availability of surgical procedures, patient and physician preferences have contributed to the increase. A large proportion of these patients (29%–48%) did not receive adequate outpatient care before surgery. For several years, the SHIs have introduced a second opinion before spinal surgery. After a second expert opinion, a large proportion of patients decided against a surgery or used alternative treatments (18). This suggests that better outpatient care and a social and medical consensus in favor of non-surgical care for patients can prevent or delay surgery. Finally, more scientific evidence is needed on how to select patients most likely to benefit from spinal surgery before increasing expensive and potentially risky invasive treatment options.

### Rehabilitation for chronic LBP

The number of rehabilitations with the main diagnosis “musculoskeletal system and connective tissue” (outpatient and inpatient rehabilitation combined) was stable, with fluctuations over the period. The fluctuations are due to a decrease in inpatient rehabilitation compensated by increased outpatient rehabilitation as a result of changes in social legislation (introduction of book IX of the social code) in 2020. We did not find any studies on the temporal trend of inpatient and outpatient rehabilitation in other countries, but as there is a general trend in health care towards shorter hospital stays, more outpatient procedures and a focus on patient progress is assumed (96, 97).

Apart from rehabilitation clinics, multimodal pain therapy for chronic LBP is provided in pain clinics covered by SHI. There is an overlap with rehabilitation services for people of working age, which limits reliable assessment of patients receiving multimodal therapy. About 5% of patients hospitalized for LBP receive multimodal pain therapy (35). Multimodal pain therapy is not a treatment option for newly diagnosed patients with LBP, which explains the low proportion of patients treated (98). Due to the different funding and responsibilities for drug treatment by the SHI and for rehabilitation by the pension insurance funds, no combined data sets are available. This precludes the analysis of treatment before and after rehabilitation for LBP. Better knowledge of the course of treatment could help to provide more targeted treatment for people at risk of losing their ability to work.

### Strength and limitations

This is the first comprehensive review of health service utilization for LBP in Germany based on claims data that allows the description of temporal trends. We identified 76 publications based on claims data and included claims data from 2 online databases. A major strength of claims data is their availability and that they cover large populations. Our aim was to describe the available evidence thus, in line with the PRISMA-ScR checklist, we did not analyze the risk of bias of each publication and claims data report. In general, claims data are of high external validity as they are a close representation of the national population. Nevertheless, the results presented must be interpreted with caution due to limited internal validity and data comparability. This is because:

• there is no existing consensus on which ICD-10 codes should be used to define LBP (Table 2).
• the ICD-10 classification does not adequately reflect the clinical problems (71).
• different standardization of data.
• no standard of the reference population and time periods.
• temporal trends and influences, such as remuneration, have to be considered.
• temporal and regional trends in utilization can only be partially captured.

The included health reports and publications used different ICD-10 codes for the classification of LBP. Assuming that pain in the lumbosacral region is the most common, we considered all codes related to LBP. Therefore, there may have been a slight overestimation of health service utilization due to misclassification (24, 71).

We are aware that there are many people who seek care outside SHI, which does not cover regularly popular services like osteopathy and that many people pay out of pocket for massage, acupuncture and other services including medication or devices. Since this data is not collected, we underestimate the use of some health services for LBP.

We did not include data on invasive non-operative therapies (injection therapies). They are underestimated by claims data as not all injection therapies can be billed. Nevertheless, as indicated by one publication, there was an increase from 0.2% to 0.6% in the utilization of injection therapy in outpatients from 2006 to 2010 (21). We do not report data on NSAIDs, which are frequently utilized for the management of LBP, because they are available over-the-counter.

We did not find any data on privately insured patients, which are approximately 10% of the German population. On average, this is a group of healthier and younger patients with a higher socio-economic status.

There is no established methodology for searching the grey literature. Nevertheless, it seems unlikely that substantial publications may have been missed, as the number of institutions publishing claims data is limited. Although the data extraction was conducted by one author, we believe that any resulting bias would be minimal, since all authors wrote specific chapters of this manuscript and re-read the original references.

## Conclusions

Most of the available German claims data cannot be pooled or compared directly because of different standardization methods or lack of standardization, different inclusion criteria, and different reference systems used for reporting. Consequently, the data provide only an incomplete picture of the health care for LBP. Given the epidemiologic and economic importance of LBP and the dynamic nature of health care delivery, a consensus on data reporting is needed to allow pooling and comparison of claims data and to support data-based decision-making. The available data on imaging, hospital admissions, and spinal surgery, despite inherent limitations, suggest an overuse of health care resources for LBP in Germany. Similar trends have been observed in other industrialized countries, warranting further investigation and quality improvement measures to optimize care delivery and resource allocation.

## Data Availability

The original contributions presented in the study are included in the article/Supplementary Material, further inquiries can be directed to the corresponding author.
