# Supplementary material for: Health service utilization for low back pain in Germany between 2000 and 2020—a scoping review of claims data
Source: Front Pain Res (Lausanne). 2025 Sep 16;6:1661722. doi: 10.3389/fpain.2025.1661722 (PMC12479410; doi:10.3389/fpain.2025.1661722)
Supplement: Supplementary file 1 [file Datasheet1.docx]

**Additional file 1: Search strategy and Health Reports of Statutory Health Insurances included**

**PubMed Search Strategy**

1. ("diagnosis"[Mesh] AND "Back Pain"[Mesh]) AND "Germany"[Mesh] AND (("2019/09/09"[PDAT] : "2025/04/09"[PDAT]) AND "humans"[MeSH Terms])
2. (("Diagnosis"[Mesh] AND "diagnostic imaging"[Subheading]) AND "Back Pain"[Mesh]) AND "Germany"[Mesh] AND (("2019/09/09"[PDAT] : "2025/04/08"[PDAT]) AND "humans"[MeSH Terms])
3. "Back Pain/drug therapy"[Mesh] AND "Germany"[Mesh] AND ((„2019/09/09“[PDAT] : „2025/04/08“[PDAT]) AND "humans"[MeSH Terms])
4. "Back Pain/economics"[Mesh] AND "Germany"[Mesh] AND ((„2019/09/09“[PDAT] : „2025/04/08“[PDAT]) AND "humans"[MeSH Terms])
5. "Back Pain/epidemiology"[Mesh] AND "Germany"[Mesh] AND ((„2019/09/09“[PDAT] : „2025/04/08“[PDAT]) AND "humans"[MeSH Terms])
6. ("Back Pain"[Mesh] AND "Patient Acceptance of Health Care"[Mesh]) AND "Germany"[Mesh] AND ((„2019/09/09“[PDAT] : „2025/04/08“[PDAT]) AND "humans"[MeSH Terms])
7. "Back Pain/rehabilitation"[Mesh] AND "germany"[MeSH Terms] AND ((„2019/09/09“[PDAT] : „2025/04/08“[PDAT]) AND "humans"[MeSH Terms])
8. "Back Pain/statistics and numerical data"[Mesh] AND "Germany"[Mesh] AND ((„2019/09/09“[PDAT] : „2025/04/08“[PDAT]) AND "humans"[MeSH Terms])
9. "Back Pain/surgery"[Mesh] AND "Germany"[Mesh] AND ((„2019/09/09“[PDAT] : „2025/04/08“[PDAT]) AND "humans"[MeSH Terms])
10. "Back Pain/therapy"[Mesh] AND "Germany"[Mesh] AND ((„2019/09/09“[PDAT] : „2025/04/08“[PDAT]) AND "humans"[MeSH Terms])

**EMBASE search strategy:**

1. 'low back pain' AND 'claims data' AND 'germany'

2. 'low back pain'/de AND diagnosis:ab AND 'germany':ab

3. 'low back pain'/de AND 'drug':ab AND 'germany':ab

4. 'low back pain'/de AND economics:ab AND 'germany':ab

5. 'low back pain'/de AND epidemiology:ab AND 'germany':ab

6. 'low back pain'/de AND rehabilitation:ab AND 'germany':ab

7. 'low back pain'/de AND surgery:ab AND 'germany':ab

8. 'low back pain'/de AND therapy:ab AND 'germany':ab

**BKK (n=8)**

**Health Reports of Statutory Health Insurances included**

1. BKK Gesundheitsreport 2003 - Gesundheit und Arbeitswelt. Essen; 03.2002.

2. BKK Gesundheitsreport 2005 - Krankheitsentwicklung - Blickpunkt: Psychische Gesundheit. Essen; 11.2005.

3. spectrumIK GmbH. BKK Gesundheitsreport 2009 - Gesundheit in Zeiten der Krise. Berlin; 12.2009.

4. spectrumIK GmbH. BKK Gesundheitsreport 2010 - Gesundheit in einer älter werdenden Gesellschaft. Berlin; 11.2010.

5. BKK Gesundheitsreport 2008 - Seelische Krankheiten prägen das Krankheitsgeschehen. Essen; 10.2008.

6. BKK Gesundheitsreport 2007 - Gesundheit in Zeiten der Globalisierung. Essen; 11.2007.

7. BKK Gesundheitsreport - Deografischer und wirtschaftlicher Wandel - gesundheitliche Folgen. Essen; 09.2006.

8. BKK Gesundheitsreport 2004 - Gesundheit und sozialer Wandel. Essen; 11.2004.

**DAK (n=11)**

9. IGES Institut GmbH. Gesundheitsreport 2014.: Die Rushhour des Lebens. Gesundheit im Spannungsfeld von Job, Karriere und Familie; DAK Gesundheitsreport 2014.

10. IGES Institut GmbH. DAK Gesundheitsreport 2011; 02.2011.

11. IGES Institut für Gesundheits- und Sozialforschung GmbH. Gesundheitsreport 2008 - Analyse der Arbeitsunfähigkeitsdaten. Schwerpunkt Mann und Gesundheit.: DAK Gesundheitsreport 2008; 02.2008.

12. IGES Institut GmbH. Gesundheitsreport 2009. Analyse der Arbeitsunfähigkeitsdaten. Schwerpunkt Doping am Arbeitsplatz: DAK Gesundheitsreport 2009; 02.2009.

13. IGES Institut GmbH. DAK Gesundheitsreport 2010; 02.2010.

14. IGES Institut GmbH. DAK-Gesundheitsreport 2012. Hamburg; 02.2012.

15. IGES Institut GmbH. DAK-Gesundheitsreport 2013. Hamburg; 02.2013.

16. IGES Institut GmbH. DAK-Gesundheitsreport 2015. Heidelberg; 03.2015.

17. Jörg Marschall, Susanne Hildebrandt, Hanna Sydow, Hans-Dieter Nolting. Gesundheitsreport 2016 Analyse der Arbeitsfähigkeitsdaten.: Schwerpunkt: Gender und Gesundheit. Heidelberg: medhochzwei Verlag GmbH; 2016. (Beiträge zur Gesundheitsökonomie und VersorgungsforschungBand 13).

18. Jörg Marschall, Susanne Hildebrandt, Hanna Sydow, Hans-Dieter Nolting. Gesundheitsreport 2017 Analyse der Arbeitsunfähigkeitsdaten: Update: Schlafstörungen. Heidelberg: medhochwzwei Verlag GmbH; 2017. (Beiträge zur Gesundheitsökonomie und VersorgungsforschungBand 16).

19. Jörg Marschall, Susanne Hildebrandt, Karsten Zich, Throsten Tisch, Jelena Sörensen, Hans-Dieter Nolting. Gesundheitsreport 2018 Analyse der Arbeitsunfähigkeitsdaten: Update: Rückenerkrankungen. Heidelberg: medhochwzwei Verlag GmbH; 2018. (Beiträge zur Gesundheitsökonomie und VersorgungsforschungBand 21).

TK (n=17)

20. Thomas Grobe, ISEG Institut für Sozialmedizin, Epidemiologie und Gesundheitssystemforschung. Gesundheitsreprot 2013 - Berufstätigkeit, Ausbildung und Gesundheit: Gesundheitsreport 2013 - Veröffentlichungen zum Betrieblichen gesundheitsmanagement der TK. Hamburg.

21. Thomas Grobe, AQUA - Institut für angewandte Qualitätsforschung im Gesundheitswesen GmbH. Gesundheitsreport 2014 - Risiko Rücken: Gesundheitsreport 2014 - Veröffentlichung zum Betrieblichen Gesundheitsmanagement der TK. Hamburg.

22. Thomas Grobe, Hans Dörning, ISEG Institut für Sozialmedizin, Epidemiologie und Gesundheitssystemforschung. Gesundheitsreport - Auswertung 2002 Arbeitsunfähigkeiten und Arzneimittelverordnung Schwerpunkt: depressive Erkrankungen: Veröffentlichung zum Betrieblichen Gesundheitsmanagement der TK. Hamburg.

23. Thomas Grobe, Hans Dörning, ISEG Institut für Sozialmedizin, Epidemiologie und Gesundheitssystemforschung. Gesundheitsreport - Auswertung 2005 Teil 1: Arbeitsunfähigkeiten Schwerpunkt: Trends: Veröffentlichung zum Betrieblichen Gesundheitsmanagement der TK. Hamburg.

24. Thomas Grobe, Hans Dörning, ISEG Institut für Sozialmedizin, Epidemiologie und Gesundheitssystemforschung. Gesundheitsreport - Auswertungen 2000 - 2001 Arbeitsunfähigkeiten und Arzneimittelversorgung: Veröffentlichung zum Betrieblichen Gesundheitsmanagement der TK. Hamburg.

25. Thomas Grobe, Hans Dörning, ISEG Institut für Sozialmedizin, Epidemiologie und Gesundheitssystemforschung. Gesundheitsreport - Auswertungen 2004 Arbeitsunfähigkeiten und Arzneimittelverordnungen Schwerpunkt: Arzneiverordnungdaten als Gesundheitsindikation: Veröffentlichung zum Betrieblichen Gesundheitsmanagement der TK. Hamburg.

26. Thomas Grobe, Hans Dörning, ISEG Institut für Sozialmedizin, Epidemiologie und Gesundheitssystemforschung. Gesundheitsreport - Auswertungen 2006 Arbeitsunfähigeiten und Arzneiverordnung Schwerpunkt: erkältungskrankheiten - Grippe: Gesundheitsreport 2006 - Veröffentlichung zum Betrieblichen Gesundheitsmanagement der TK. Hamburg.

27. Thomas Grobe, Hans Dörning, ISEG Institut für Sozialmedizin, Epidemiologie und Gesundheitssystemforschung. Gesundheitsreport - Auswertungen 2007 Arbeitsunfähigkeiten und Arzneimittelverordnungen Schwerpunkt: Gesundheit im demografischen Wandel: Gesundheitsreport 2007 - Veröffentlichung zum betrieblichen Gesundheitsmanagement der TK.

28. Thomas Grobe, Hans Dörning, ISEG Institut für Sozialmedizin, Epidemiologie und Gesundheitssystemforschung. Gesundheitsreport - Auswertungen 2008 Arbeitsunfähigkeiten und Arzneimittelverordnungen Schwerpunkt: Psychische Störung: Gsundheitsreport 2008 - Veröffentichung zum Betrieblichen Gesundheitsmanagemnet der TK. Hamburg.

29. Thomas Grobe, Hans Dörning, ISEG Institut für Sozialmedizin, Epidemiologie und Gesundheitssystemforschung. Gesundheitsreport - Auswertungen 2009 Arbeitsunfähigkeiten und Arzneimittelverordnungen Schwerpunkt: Gesundheit von Beschäftigten in Zeitarbeitsunternehmen: Gesundheitsreport 2009 - Veröffentlichungen zum betrieblichen Gesundheitsmanagement der TK. Hamburg.

30. Thomas Grobe, Hans Dörning, ISEG Institut für Sozialmedizin, Epidemiologie und Gesundheitssystemforschung. Gesundheitsreport 2010 - Gesundheitliche Veränderungen bei Berufstätigen und Arbeitslosen von 2000 bis 2009: Gesundheitsreport 2010 - Veröffentlichungen zum Betrieblichen Gesundheitsmanagement der TK. Hamburg.

31. Thomas Grobe, Hans Dörning, ISEG Institut für Sozialmedizin, Epidemiologie und Gesundheitssystemforschung. Gesundheitsreport 2011 - Gesundheitliche Veränderungen bei jungen Erwerbspersonen und Studierenden: Gesundheitsreport 2011 - Veröffentlichungen zum Betrieblichen Gesundheitsmanagement der TK. Hamburg.

32. Thomas Grobe, ISEG Institut für Sozialmedizin, Epidemiologie und Gesundheitssystemforschung. Gesundheitsreport 2012 - Mobilität, Flexibilität, Gesundheit: Gesundheitsreport 2012 - Veröffentlichungen zum Betrieblichen Gesundheitsmanagement der TK.

33. Thomas Grobe, Susanne Steinmann, AQUA - Institut für angewandte Qualitätsförderung und Forschung im Gesundheitswesen GmbH. Gesundheitsreport 2015 - Gesundheit von Studierenden: Gesundheitsreport 2015 - Veröffentlichungen zum Betrieblichen Gesundheitsmanagement der TK. Hamburg.

34. Thomas Grobe, Susanne Steinmann, AQUA - Institut für angewandte Qualitätsförderung und Forschung im Gesundheitswesen GmbH. Gesundheitsreport 2016 - Gesundheit zwischen Beruf und Familie: Gesundheitsreport 2016 - Veröffentlichung zum Betrieblichen Gesundheitsmanagement der TK.

35. Thomas Grobe, Susanne Steinmann, Julia Gerr, AQUA - Institut für angewandte Qualitätsförderung und Forschung im Gesundheitswesen GmbH. Gesundheitsreport - Arbeitsunfähigeiten. Hamburg.

36. Thomas Grobe, Susanne Steinmann, Julia Gerr, AQUA - Institut für angewandte Qualitätsförderung und Forschung im Gesundheitswesen GmbH. Gesundheitsreport - Weitere Auswertung zu Arbeitsunfähigkeiten. Hamburg.

**Barmer/GEK (n=23)**

37. Deitermann B., Kemper C., Hoffmann F., Glaeske G. GEK-Heil- und Hilfsmittel-Report 2006: Auswertungsergebnisse der GEK-Heil- und Hilfmitteldaten aus den Jahren 2004 und 2005. Sankt Augustin: Asgard. GEK-Heil- und Hilfsmittel-Report : Auswertungsergebnisse der GEK-Heil- und Hilfsmitteldaten aus den Jahren …; 2006. (GEK-Edition; vol 49).

38. Grandt D., Kossack N., Brechtel T. BARMER Heil- und Hilfsmittelreport 2017: Schriftenreihe zur Gesundheitsanalyse. Siegburg: Ansgard Verlagsservice GmbH. Heil- und Hilfsmittelreport …; 2017. (Schriftenreihe zur GesundheitsanalyseBand 6).

39. Bitzer EM, Grobe TG, Neusser S, Miethe I, Schwartz FW. BARMER GEK Report Krankenhause 2011: Schwerpunktthema: Der Übergang von der stationären zur ambulanten Versorgung bei psychischen Störungen. St. Augustin: Asgard-Verl.; 2011. (Schriftenreihe zur Gesundheitsanalyse; vol 9).

40. Augurzky B, Fels K, Pilny A, Wübker A, Bohms, Priess H.-W. et al. BARMER GEK Report Krankenhaus 2016: Auswertung zu Daten bis 2015 Schwerpunkt: Adipositas. Siegburg: Asgard Verlagsservice GmbH; 2016. (Schriftenreihe zur Gesundheitsanalyse; vol 40).

41. Bitzer EM, Grobe TG, Schilling E., Dörning H, Schwartz FW. GEK-Report Krankenhaus 2009: Schwerpunktthema: Alkoholmissbrauch bei Jugendlichen. St. Augustin: Asgard-Verl.; 2009. (Schriftenreihe zur Gesundheitsanalyse; vol 69).

42. Augurzky B, Hentschker C, Pilny A, Wübker A. BARMER Krankenhausreport 2017. Siegburg: Asgard-Verlagsservice GmbH. Krankenhausreport …; 2017. (Schriftenreihe zur GesundheitsanalyseBand 4).

43. Bitzer EM, Lehmann B, Bohm S, Priess H.-W. BARMER GEK Report Krankenhaus 2015: Auswertung zu Daten bis 2014 Schwerpunkt: Lumbale Rückenschmerzen. Siegburg: Asgard-Verl.-Service; 2015. (Schriftenreihe zur Gesundheitsanalyse; vol 33).

44. Bitzer EM, Grobe TG, Neusser S., Dörning H, Schwartz FW. GEK-Report akut-stationäre Versorgung 2008: Schwerpunktthema: Kinder im Krankenhaus. Sankt Augustin: Asgard-Verl.; 2008. (Schriftenreihe zur Gesundheitsanalyse; vol 63).

45. Bitzer EM, Grobe TG, Neusser S, Schneider A, Dörning H, Schwartz FW. Barmer GEK Report Krankenhaus 2010: Schwerpunktthema: Trends in der Endoprothetik des Hüft- und Kniegelenks. St. Augustin: Asgard-Verl.; 2010. (Schriftenreihe zur Gesundheitsanalyse; vol 3).

46. Bitzer EM, Grobe TG, Neusser S, Lorenz C. BARMER GEK Report Krankenhaus 2013: Schwerpunktthema: Die stationäre Versorgung von Darmkrebs. Siegburg: Asgard-Verl.-Service; 2013. (Schriftenreihe zur Gesundheitsanalyse; vol 21).

47. Bitzer EM, Grobe TG, Dörning H, Schwartz FW. GEK-Report akut-stationäre Versorgung 2006. St. Augustin: Asgard-Verl.; 2006. (Schriftenreihe zur GesundheitsanalyseBd. 45).

48. Bitzer EM, Grobe TG, Neusser S, Lorenz C, Schwartz FW. BARMER GEK Report Krankenhaus 2012: Schwerpunktthema: die akut-stationäre Versorgung des Prostatakarzinoms. Siegburg: Asgard-Verl.-Service; 2012. (Schriftenreihe zur Gesundheitsanalyse; vol 15).

49. Bitzer EM, Grobe TG, Dörning H, Schwartz FW. GEK Report akut-stationäre Versorgung 2007: Thema: Koronare Herzkrankheit - Interventionelle Kardiologie und Herzchirurgie. St. Augustin: Asgard-Verl.; 2007. (Schriftenreihe zur Gesundheitsanalyse; vol 58).

50. Bitzer EM, Bohm S, Hartmann A, Priess H.-W. BARMER GEK Report Krankenhaus 2014: Schwerpunktthema: Die Trends in der koronaren Revaskularisation. Siegburg: Asgard-Verl.-Service; 2014. (Schriftenreihe zur Gesundheitsanalyse; vol 27).

51. Grobe TG, Dörning H, Schwartz FW. Barmer GEK Arztreport: Auswertung zu Daten bis 2008 Schwerpunkt: Erkrankungen und zukünftige Ausgaben. St. Augustin: Asgard-Verl.; 2010. (Schriftenreihe zur Gesundheitsanalyse; vol 1).

52. Grobe TG, Dörning H, Schwartz FW. Schwerpunkt: Bildgebende Diagnostik - Computer- und Magnetresonanztomographie. St. Augustin: Asgard-Verl.; 2011. (Schriftenreihe zur Gesundheitsanalyse; vol 6).

53. Grobe TG, Dörning H, Schwartz FW. Barmer GEK Arztreport 2012: Schwerpunkt: Kindergesundheit. St. Augustin: Asgard-Verl.; 2012. (Schriftenreihe zur Gesundheitsanalyse; vol 12).

54. Grobe TG, Bitzer EM, Schwartz FW. BARMER GEK Arztreport 2013: Schwerpunkt: Aufmerksamkeitsdefizit-/Hyperaktivitätsstörungen ADHS. Siegburg: Asgard-Verl.-Service; 2013. (Schriftenreihe zur Gesundheitsanalyse; vol 18).

55. Grobe TG, Heller G, Szecsenyi J. Barmer GEK Arztreport 2014: Schwerpunkt: Hautkrebs. Siegburg: Asgard-Verl.-Service; 2014. (Schriftenreihe zur Gesundheitsanalyse; vol 24).

56. Grobe TG, Klingenberg A, Szecsenyi J, Steinmann S. Barmer GEK Arztreport 2015: Schwerpunkt: Gebärmutterhalskrebs. Siegburg: Asgard-Verl.-Service; 2015. (Schriftenreihe zur Gesundheitsanalyse; vol 30).

57. Grobe TG, Steinmann S, Szecsenyi J. BARMER GEK Arztreport 2016: Schwerpunkt: Alter und Schmerz. Siegburg: Asgard-Verlagsservice GmbH; 2016. (Schriftenreihe zur Gesundheitsanalyse; vol 37).

58. Grobe TG, Steinmann S, Szecsenyi J. BARMER Arztreport 2017: Schriftenreihe zur Gesundheitsanalyse. Siegburg: Asgard-Verlagsservice GmbH. Arztreport …; 2017. (Schriftenreihe zur GesundheitsanalyseBand 1).

59. Grobe TG, Steinmann S, Szecsenyi J. BARMER Arztreport 2018: Schriftenreihe zur Gesundheitsanalyse. Siegburg: Asgard-Verlagsservice GmbH. Arztreport …; 2018. (Schriftenreihe zur GesundheitsanalyseBand 7).

**AOK (n=5)**

60. Waltersbacher A. Heilmittelbericht 2018: Ergotherapie Sprachtherapie Physiotherapie Podologie.

61. Waltersbacher A. Heilmittelbericht 2019 Ergotherapie, Sprachtherapie, Physiotherapie, Podologie; 2019.

62. Waltersbacher A. Heilmittelbericht 2017: Ergotherapie Sprachtherapie Physiotherapie Podologie; 2017 2017.

63. Waltersbacher A. Heilmittelbericht 2016: Ergotherapie Sprachtherapie Physiotherapie Podologie; 2016.

64. Klauber/Günster/Robra/Schmacke, editor. Versorgungs-Report 2013/2014; 2014.
