# Supplementary material for: Health service utilization for low back pain in Germany between 2000 and 2020—a scoping review of claims data
Source: Front Pain Res (Lausanne). 2025 Sep 16;6:1661722. doi: 10.3389/fpain.2025.1661722 (PMC12479410; doi:10.3389/fpain.2025.1661722)
Supplement: Supplementary file 2 [file Table1.docx]

**Appendix 2: Overview over extracted data from health reports of statutory health insurances, federal health monitoring, federal statistical office and single publications**

|  | **Format of data extracted** | **Standardization** | **Information on regional differences** |
| --- | --- | --- | --- |
| Ambulatory Consultation | % of insured persons,  number of treated cases per 1000 insured people | no standardization (DAK data)  standardization with different methods (AOK, DEWI report (1)) | yes (2) |
| Imaging | % of insured persons | standardized for age, sex and region (3) | yes (3) |
| Invasive non-operative therapies | % of insured persons | data standardized for age and sex of the general population (2) | no |
| Non-pharmaceutical prescription treatments | % of insured persons with diagnosis of LBP | no standardization or  standardization with different methods | no |
| Non-invasive therapies | % of insured persons with diagnosis of LBP | data standardized for age and sex since health report year 2013 (4) | no |
| Prescription medication | % of insured persons with diagnosis of LBP | no (5) | no |
| Sick leave days | Days of sick leave per 100 insured persons | data standardized for age and sex since health report year 2013 | no |
| Hospitalization | Cases per 10,000 insurance years  Cases per 100,000 residents | data standardized for age and sex of the general population (2) | Data available for all 16 federal states |
| Spine surgery | % of persons hospitalized with low back pain | data standardized for age and sex (6) | Data available for all 16 federal states (6) |
| Rehabilitation | Number of rehabilitations | not applicable | no |

Literature Cited

1. Jochen Schmitt, Falko Tesch, Toni Lange. Determinanten bei der Versorgung von Patienten mit Wirbelsäulenoperation (DEWI). Available from: URL: https://innovationsfonds.g-ba.de/downloads/beschluss-dokumente/4/2020-12-18_DEWI_Ergebnisbericht.pdf.

2. Chenot J, Haupt C, Gerste B. Zeitliche Trends bei der Versorgung von Rückenschmerzpatienten. In: Klauber/Günster/Robra/Schmacke, editor. Versorgungs-Report 2013/2014; 2014. p. 155–83.

3. Andersohn F WJ. Faktencheck Rücken: Ausmaß und regionale Variationen von Behandlungsfällen und bildgebener Diagnostik; 2016.

4. Hickstein L, Kiel S, Raus C, Heß S, Walker J, Chenot J-F. Akupunktur als Leistung der gesetzlichen Krankenversicherung in Deutschland : Eine retrospektive Beobachtungstudie basierend auf Abrechnungsdaten 1.

5. Daniel T, Koetsenruijter J, Wensing M, Wronski P. Chronische Kreuzschmerzen – Nutzertypen ambulanter Versorgung : Eine Clusteranalyse der Inanspruchnahme ambulanter Regelversorgung anhand von Routinedaten der AOK Baden-Württemberg. Schmerz 2022; 36(5):326–32.

6. Zich K Tisch T. Faktencheck Rücken Rückenschmerzbedingte Krankenhausaufenthalte und operative Eingriffe – Mengenentwicklung und regionale Unterschiede; 2017.
